# Atmospheric chemosynthesis is phylogenetically and geographically widespread and contributes significantly to carbon fixation throughout cold deserts

**DOI:** 10.1038/s41396-022-01298-5

**Published:** 2022-08-06

**Authors:** Angelique E. Ray, Julian Zaugg, Nicole Benaud, Devan S. Chelliah, Sean Bay, Hon Lun Wong, Pok Man Leung, Mukan Ji, Aleks Terauds, Kate Montgomery, Chris Greening, Don A. Cowan, Weidong Kong, Timothy J. Williams, Philip Hugenholtz, Belinda C. Ferrari

**Affiliations:** 1 School of Biotechnology and Biomolecular Sciences, UNSW, Sydney, NSW 2052 Australia; 2 School of Chemistry and Molecular Biosciences, Australian Centre for Ecogenomics, The University of Queensland, St Lucia, QLD 4072 Australia; 3 School of Biological Sciences, Monash University, Clayton, VIC 3800 Australia; 4 Department of Aquatic Microbial Ecology, Institute of Hydrobiology, Biology Centre of the Academy of Sciences of the Czech Republic, České Budějovice, Czech Republic; 5 Key Laboratory of Alpine Ecology, Institute of Tibetan Plateau Research, Chinese Academy of Sciences, 100101 Beijing, China; 6 Center for Pan-Third Pole Environment, Lanzhou University, Lanzhou, 730000 China; 7 Australian Antarctic Division, Department of Environment, Antarctic Conservation and Management, Kingston, TAS Australia; 8 Centre for Microbial Ecology and Genomics, Department of Biochemistry, Genetics and Microbiology, University of Pretoria, Pretoria, 0002 South Africa

**Keywords:** Soil microbiology, Microbial ecology, Microbial genetics, Metagenomics, Microbiome

## Abstract

Cold desert soil microbiomes thrive despite severe moisture and nutrient limitations. In Eastern Antarctic soils, bacterial primary production is supported by trace gas oxidation and the light-independent RuBisCO form IE. This study aims to determine if atmospheric chemosynthesis is widespread within Antarctic, Arctic and Tibetan cold deserts, to identify the breadth of trace gas chemosynthetic taxa and to further characterize the genetic determinants of this process. H_2_ oxidation was ubiquitous, far exceeding rates reported to fulfill the maintenance needs of similarly structured edaphic microbiomes. Atmospheric chemosynthesis occurred globally, contributing significantly (*p* < 0.05) to carbon fixation in Antarctica and the high Arctic. Taxonomic and functional analyses were performed upon 18 cold desert metagenomes, 230 dereplicated medium-to-high-quality derived metagenome-assembled genomes (MAGs) and an additional 24,080 publicly available genomes. Hydrogenotrophic and carboxydotrophic growth markers were widespread. RuBisCO IE was discovered to co-occur alongside trace gas oxidation enzymes in representative *Chloroflexota*, *Firmicutes*, *Deinococcota* and *Verrucomicrobiota* genomes. We identify a novel group of high-affinity [NiFe]-hydrogenases, group 1m, through phylogenetics, gene structure analysis and homology modeling, and reveal substantial genetic diversity within RuBisCO form IE (*rbcL1E*), and high-affinity 1h and 1l [NiFe]-hydrogenase groups. We conclude that atmospheric chemosynthesis is a globally-distributed phenomenon, extending throughout cold deserts, with significant implications for the global carbon cycle and bacterial survival within environmental reservoirs.

## Introduction

Microbial communities inhabiting cold desert soils thrive [1–5] despite limited exposure to liquid water, scarce organic and inorganic edaphic resources, frequent freeze-thaw cycles and, particularly in the polar latitudes, extreme and variable exposure to sunlight and UV radiation [6–9]. Polar desert soils are usually oligotrophic and often contain limited photosynthetic taxa, resulting in a need to supplement energy and organic carbon inputs through alternative autotrophic processes beyond photosynthesis and geochemically driven chemoautotrophy [5, 10–12]. The microbial oxidation of atmospheric molecular hydrogen (H_2_) and carbon monoxide (CO) gases are well known processes that provide soil bacteria with a reliable and ubiquitous source of energy to persist [13–21]. However, the oxidation of these trace gases has also been linked to an under-investigated chemoautotrophic primary production process known as atmospheric chemosynthesis, which is hypothesized to support microbial carbon inputs in extreme terrestrial environments where photosynthetic input is low [11, 22–25]. During atmospheric chemosynthesis, trace levels of CO and H_2_ are oxidized using type 1 [MoCu]-CO dehydrogenases (CODH) and high-affinity group 1h [NiFe]-hydrogenases (*hhyL*), respectively. Additional high-affinity hydrogenases, specifically group 1l [NiFe]-hydrogenase (*hylL*) [22, 24] and group 2a [NiFe]-hydrogenase (*hucL*) [17], have recently been discovered that may also contribute to this process. Electrons yielded from atmospheric H_2_ and CO oxidation are proposed to act in conjunction with ribulose-1,5-biphosphate carboxylase/oxygenase (RuBisCO) to support carbon fixation through the Calvin-Benson-Bassham (CBB) cycle [11, 22]. The novel RuBisCO form IE is the major form associated with atmospheric chemosynthesis, due to its high relative abundance within microbiomes where atmospheric chemosynthetic activity has been observed [11]. RuBisCO form IE is phylogenetically related to the light-independent RuBisCO forms IC and ID [26, 27]. The genes encoding RuBisCO form IE (*rbcL1E*) and high-affinity hydrogenase (*hhySL*) are ubiquitous and in high abundance across oligotrophic deserts spanning the Antarctic, Arctic and Tibetan Plateau [12], yet activity studies confirming atmospheric chemosynthesis outside niche communities in eastern Antarctica are lacking. While 19 bacterial and six archaeal phyla contain trace gas oxidizers that use the energy derived from aerobic respiration to support persistence [22, 28], thus far only three phyla have been proposed to be capable of atmospheric chemosynthesis: *Actinobacteriota*, *Candidatus* Dormibacterota and *Ca*. Eremiobacterota [11].

We hypothesize that atmospheric chemosynthesis is a globally-distributed phenomenon, occurring widely in cold edaphic niches where photosynthetic capabilities are limited and in a broad range of taxa common throughout these environments. We use metagenomics, phylogenetics and biochemical approaches to quantify the contribution of atmospheric chemosynthesis to primary production in soils from cold deserts that span the globe, including New Harbour (NH), Taylor Valley, Mitchell Peninsula (MP), Windmill Islands and The Ridge (TR), Vestfold Hills, in Antarctica; the Ngari Prefecture in the Qinghai-Tibet Plateau (TP) in China; and Spitsbergen, Svalbard (SS) and Alexandra Fjord Highlands (AFH) in the high Arctic. Physicochemical variation has been observed across all sites, capturing a range of conditions common amongst cold desert environments (average dry matter fraction = 0.763 at Alexandra Fjord Highlands—0.998 at TP, average pH = 5.41 at Mitchell Peninsula—8.87 at The Ridge, average total carbon (TC) (% w/w) = 0.08 at The Ridge—23.17 at TP (Supplementary 1) [12, 29–31]. Genome-resolved metagenomics was used to determine autotrophic capacities within our 18 soil metagenomes and 230 dereplicated MAGs, as well as 24,080 reference genomes from the Genome Taxonomy Database (GTDB) originating from a broad range of environments. We expand substantially upon the known diversity of the RuBisCO form IE gene and identify its co-occurrence with trace gas oxidation genes in representatives from seven bacterial phyla that are common throughout edaphic environments.

## Trace gas chemosynthetic phyla dominate cold desert soil microbiomes

**Fig. 1:**
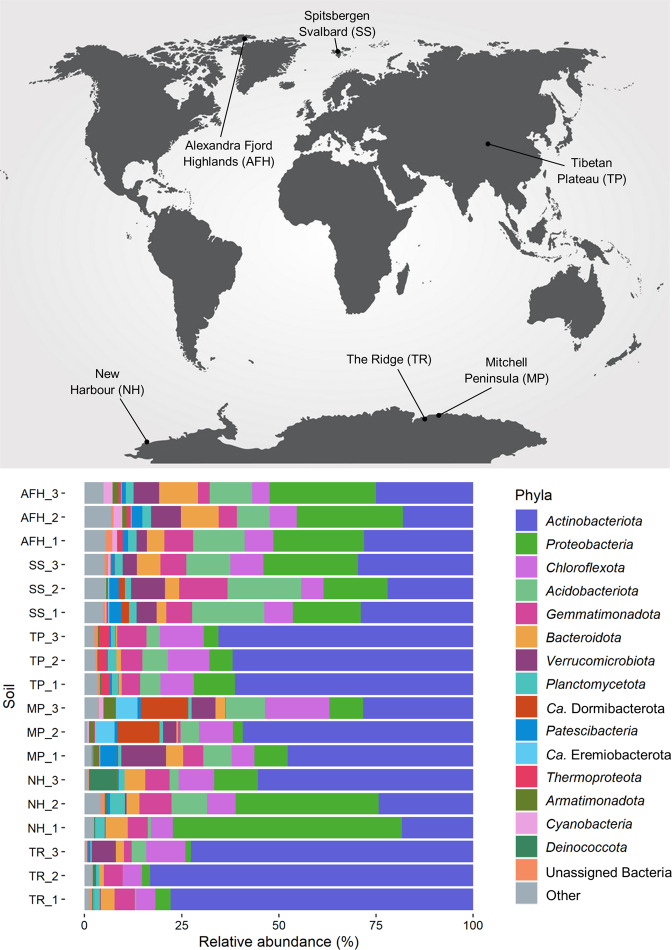
Community composition of the 18 global desert soils, classified using the universal single-copy ribosomal protein gene *rplP* retrieved from shotgun metagenomic reads. The relative abundance of major bacterial and archaeal phyla residing in triplicate desert soils from Alexandra Fjord Highlands (AFH), Spitsbergen Svalbard (SS), Tibetan Plateau (TP), Mitchell Peninsula (MP), New Harbour (NH) and The Ridge (TR) are displayed; phyla with <2% relative abundance in all soil samples were grouped to the “Other” phyla. *Actinobacteriota* dominate all sites, particularly The Ridge (average 77.9%), TP (average 62.9%) and Mitchell Peninsula (average 45.2%). Photosynthetic *Cyanobacteria* are extremely scarce within NH, TP and The Ridge samples (<0.07%), with greater average abundances observed at Mitchell Peninsula (0.6%), SS (0.4%) and Alexandra Fjord Highlands (2.0%). *Ca*. Eremiobacterota and *Ca*. Dormibacterota dominate Mitchell Peninsula microbiomes (average 7.8% and 3.6%, respectively) and are present at lower levels within SS and Alexandra Fjord Highlands. Archaea are minor members of these ecosystems (average relative abundances; <0.2% within The Ridge, Mitchell Peninsula, NH; 2.6% within TP; 0.5% within SS; 1.2% within Alexandra Fjord Highlands).

Abundant communities were detected across all sites (average 16S rRNA copy number = 3.54 × 10^8^–1.13 × 10^9^/g soil) (Supplementary 2). Shotgun sequencing produced ~8–10 Gb metagenomes from each of the 18 Antarctic, Arctic and Tibetan Plateau soil samples (Supplementary 3). Sequencing depth and coverage was assessed using a combination of rarefaction analysis and redundancy-based approaches (Supplementary 3). Antarctic soils have >70% coverage, whilst non-Antarctic samples have ~23% coverage. Analysis of prokaryotic marker genes in these metagenomes indicated that the bacterial and archaeal communities comprised 48 phyla and 127 classes (Supplementary 5). Consistent with previous studies, polar soils were dominated by *Actinobacteriota*, *Proteobacteria*, *Chloroflexota* and *Acidobacteriota*, with *Gemmatimonadota*, *Bacteroidota* and *Verrucomicrobiota* also prevalent in the High Arctic and some Antarctic sites (Fig. 1 and Supplementary 5 and 6) [5, 24, 29, 32]. Phyla associated with atmospheric chemosynthesis, including *Actinobacteriota* dominated the Antarctic and Tibetan Plateau soil samples, accounting for up to 83% of the microbial community in The Ridge, while *Ca*. Dormibacterota and *Ca*. Eremiobacterota were abundant in Mitchell Peninsula samples, accounting up to 12.3% and 5.6%, respectively according to analysis of the *rplP* marker protein (Fig. 1 and Supplementary 5). When analyzed against GTDB reference genomes, *Ca*. Dormibacterota abundances were far greater, ranging up to 49.1% of the communities at Mitchell Peninsula (Supplementary 6). By contrast, the photosynthetic *Cyanobacteria* were rare, accounting for an average relative abundance of <0.57% in soils from each Antarctic site and the Tibetan Plateau. Comparatively, *Actinobacteriota* were less abundant in high Arctic soils (18.1–29.6%) (Supplementary 5), which exhibited a higher abundance of *Cyanobacteria* compared to the Antarctic sites (Spitsbergen Svalbard 0.1–0.8% and Alexandra Fjord Highlands 1.3–2.3%) (Fig. 1 and Supplementary 5). Eukaryotic taxa were limited within all soil metagenomes (average < 0.012%) and were dominated by *Ascomycota* and *Basidiomycota* (Supplementary 7), suggesting a limited capacity for lichen formation. Photosynthetic eukaryotic phyla, specifically *Chlorophyta*, *Cryptophyta*, *Ochrophyta* and *Rhodophyta*, occurred at very low relative abundances (<0.0013%) (Supplementary 7).

Assembly and binning of all 18 metagenomes yielded 230 high or medium-quality (≥50% completeness and ≤10% contamination) MAGs after dereplication at the species level (≤95% ANI)). Of these, 76 were estimated to be more than 90% complete and <5% contaminated (Supplementary 8). The MAGs encompassed 16 bacterial and 1 archaeal phyla, with the obtained taxa reflecting metagenomic profiles with *Actinobacteriota* (*n* = 86), *Chloroflexota* (*n* = 23), *Proteobacteria* (*n* = 21), *Acidobacteriota* (*n* = 20), *Bacteroidota* (*n* = 18), *Verrucomicrobiota* (*n* = 11) and *Gemmatimonadota* (*n* = 10) being the most dominant (Supplementary 8). Archaeal MAGs were solely obtained from the phylum *Thermoproteota* (*n* = 7) (Supplementary 8). Several recently proposed bacterial candidate phyla were also represented by MAGs including *Ca*. Eremiobacterota [33], *Ca*. Dormibacterota [34], *Ca*. Sumerlaeota [35] and *Ca*. Patescibacteria [36, 37] as well as two MAGs from the metabolically flexible predatory phylum *Bdellovibrionota* (Supplementary 8) [38, 39]. MAGs spanned autotrophic phyla including those previously associated with atmospheric chemosynthesis (*Actinobacteriota*, *Ca*. Eremiobacterota and *Ca*. Dormibacterota) (*n* = 96) [11], oxygenic photosynthesis (*Cyanobacteria*) (*n* = 2) and anoxygenic photosynthesis (*Proteobacteria*: orders *Rhizobiales*, *Rhodobacterales* and *Burkholderiales*) (*n* = 6) [40]. Photoautotrophic green sulfur bacteria belonging to the class *Chlorobi* were notably undetected in both the MAGs and metagenomes, as were photoheterotrophic taxa including the genera *Ca*. Chloracidobacterium, *Heliobacterium* and *Gemmatimonas* [40–45]. *Chloroflexia*, the sole photoautotrophic class within *Chloroflexota* [46], was undetected. The only *Chloroflexota* MAGs represented belonged to the chemolithoautotrophic order *Thermomicrobiales* (*n* = 7), which is associated with CO oxidation [15, 47], and the poorly characterized candidate order 54–19 (*n* = 2).

## Photoautotrophic and geochemical-driven chemoautotrophic capacities are limited in arid and hyperarid polar soil microbiomes

**Fig. 2:**
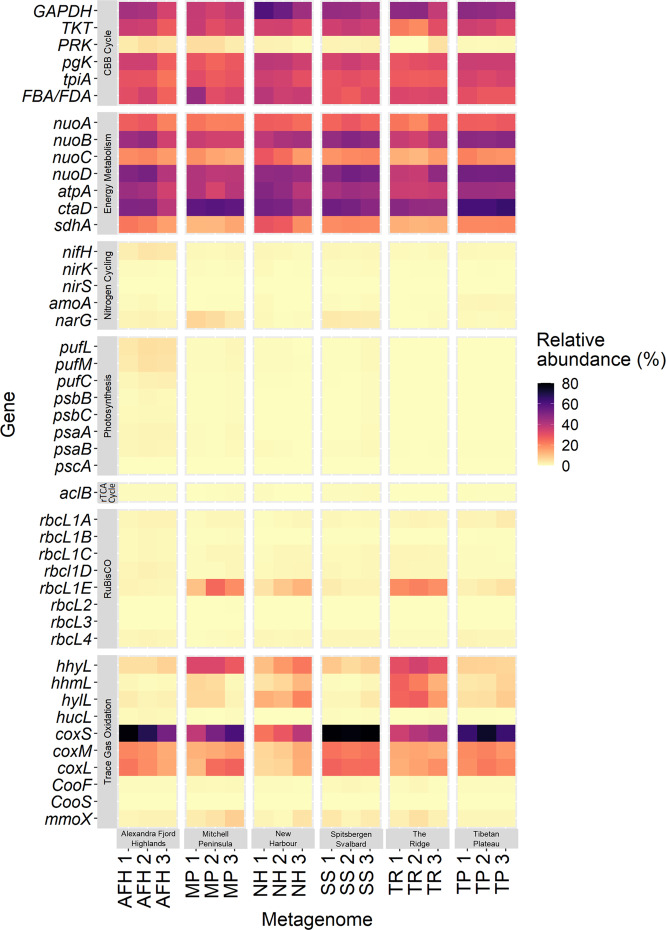
Heatmap displaying the abundance of key metabolic marker genes involved in carbon fixation and energy conservation, and their distribution throughout 18 metagenomes spanning six cold desert regions. For each region, relative gene abundances were displayed for three metagenomes. Genes encoding the CBB cycle, energy metabolism, aerobic respiration, nitrogen cycling, and trace gas oxidation were widely distributed throughout all environments. Phototrophy genes were more abundant within Alexandra Fjord Highlands metagenomes than the other sites studied.

**Fig. 3:**
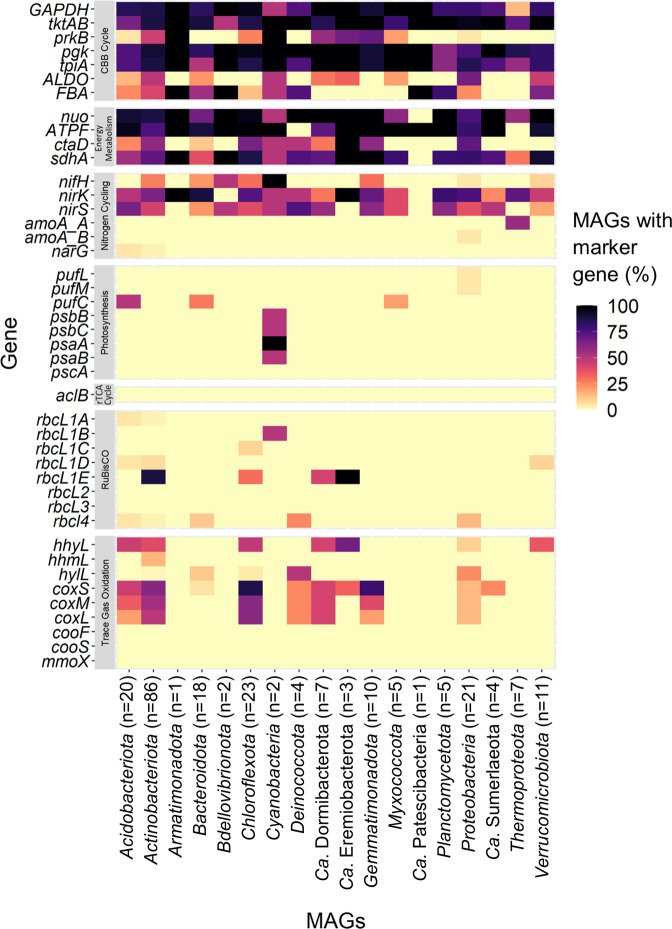
Heatmap displaying key functional genes involved in microbial autotrophy and energy conservation, and their distribution throughout the 76 high-quality (>90% completeness, <5% contamination) and 154 medium-quality bins (50–90% completeness, 5–10% contamination) constructed. Abundances are displayed relative to the total number of MAGs from each phylogenetic group. Genes encoding the CBB cycle, energy metabolism, respiration, and nitrogen cycling were widely distributed. Phototrophy genes were primarily limited to *Cyanobacteria* MAGs, whilst trace gas oxidation genes were widely distributed throughout 11 of the 18 phyla detected through MAG construction.

To understand the autotrophic strategies sustaining life in cold desert ecosystems, we explored the potential for carbon and nitrogen cycling within soil metagenomes and MAGs. Genetic markers of aerobic and anaerobic respiration were ubiquitous in samples from all six deserts and in the recovered MAGs (Figs. 2 and 3 and Supplementary 9 and 10). Genes required for lithoautotrophic processes driven by metabolizing edaphic materials were detected in a limited number of MAGs (Supplementary 10–12). Autotrophic genes detected included those associated with the oxidation of reduced inorganic sulfur compounds through the thiosulfate oxidation pathways (*Sox*) and the reverse dissimilatory sulfite reductase pathway (*dsrC*, *dsrEFH*) (Supplementary 11) [48, 49]. Together, this suggests that the MAGs recovered here have a low capacity to support microbial carbon fixation through the oxidation of geochemical substrates.

**Fig. 4:**
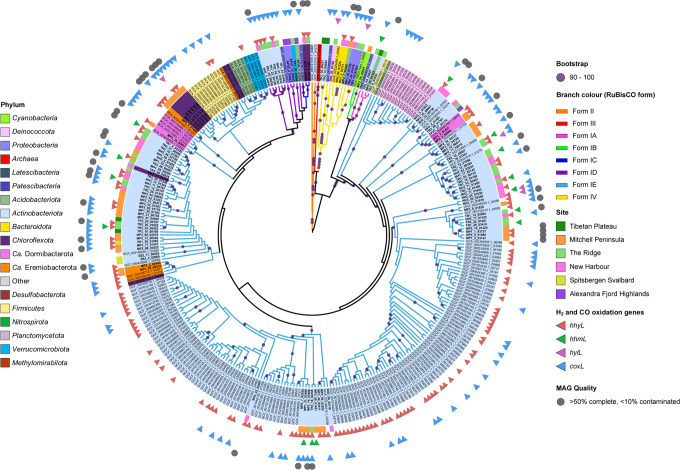
Maximum likelihood phylogenetic tree of RuBisCO gene sequences focusing on form IE, pruned from a larger tree containing binned cold desert metagenomic assembled genomes (MAGs) and over 3000 published genomes. Leaves are colored to represent phylum, while colored branches show RuBisCO form. The cold desert-site that each MAG was obtained from is shown in the outer ring. Genomes which additionally harbored high-affinity groups 1h [NiFe]-hydrogenase (*hhyL*), 1m [NiFe]-hydrogenase (*hhmL*), 1l [NiFe]-hydrogenase (*hylL*) and/or aerobic carbon monoxide dehydrogenase (*coxL*) with an active-site loop are indicated by outer triangles, colored red, green, pink, and blue, respectively. Bootstrap values >90% are depicted as filled circles on branches. Medium and high-quality MAGS constructed in this study are marked with gray circles. RuBisCO form IE is highly diverse, spanning 8 bacterial phyla (*Actinobacteriota*, *Chloroflexota*, *Firmicutes*, *Verrucomicrobiota*, *Ca*. Dormibacterota, *Ca*. Eremiobacterota, *Acidobacteriota* and *Deinococcota*) with multiple distinct clades observed. Most genomes containing RuBisCO form IE also contained high-affinity group 1 [NiFe]-hydrogenase and/or aerobic carbon monoxide dehydrogenase.

Genes associated with ammonia oxidation (*amoA*, *hao*) were uncommon and genes associated with nitrite-oxidation (*nxrAB*) were not detected in any MAGs (Supplementary 12). There was genomic evidence for biological nitrogen fixation across all six deserts. Consistent with the low *Cyanobacteria* abundances, the nitrogenase gene (*nifH)* was also low in abundance throughout the metagenomes (0.45–3.8%) (Supplementary 9). Ammonia monooxygenase (*amoA*), associated with nitrification, was uncommon in all metagenomes (<1.3%) especially in the Antarctic (average 0.13%) (Supplementary 9) and were almost exclusively detected in the ammonia oxidizing archaea (*Thermoproteota*) MAGs (Supplementary 12), a common feature in environmentally constrained Antarctic soils [29, 50–53]. Denitrification capacities were widespread, with copper-containing nitrite reductase (*nirK)* generally more prevalent than *nirS* in both the metagenomes (Supplementary 9) and MAGs (Fig. 4 and Supplementary 12). Most MAGs encode for a wide range of carbohydrate-active enzymes (CAZys) that are predicted to hydrolyze starch, hemicellulose, chitin and oligosaccharides (Supplementary 7), suggesting a capacity for organotrophy and the use of sugar-containing biopolymers as carbon and energy sources. Notably, such organic substrates are limited in oligotrophic desert soils, especially in Antarctic regions where plant matter is generally limited to moss and lichens [54, 55], consistent with the very low eukaryotic signal in all metagenomes studied here (relative average abundance <0.012%) (Supplementary 7). However, the detection of these complex carbohydrate-degrading enzymes indicates a prevalence of taxa capable of both heterotrophy and autotrophy, consistent with previous studies of terrestrial desert microbiomes [11, 22, 56, 57].

Photosynthetic markers (*psaAB*, *psbBC*, *pscA* and *pufLMC*) were detected at very low levels throughout the metagenomes (<0.93%) but occurred at higher abundances within the high Arctic site, Alexandra Fjord Highlands (<4.79%) (Supplementary 9). Genes associated with photosystem I (*psaA-F, psaI-M, psaX*) and II (*psbA-F*, *psbH-M*, *psbOP*, *psbT-Z*, *psb27*, *psb28*, *psb28-2*) were detected solely within the two *Cyanobacteria* MAGs found exclusively within Alexandra Fjord Highlands samples, whilst photosynthetic reaction center genes commonly associated with *Chlorobi* (*pscA-D*) were not detected (Supplementary 10). In comparison, anoxygenic photosynthesis genes (*pufBA-LMC*) were detected in 17 MAGs spanning the *Acidobacteriota*, *Bacteroidota*, *Myxococcota* and *Proteobacteria* (Supplementary 10). In addition to these reaction center genes, we investigated genes encoding light-harvesting complexes and antenna proteins, which play an important role in the absorption of light for photosynthesis. Phycobilisome genes were detected within a single *Chloroflexota* MAG present in Mitchell Peninsula soil (average 4.13%) and in both *Cyanobacteria* MAGs present in Alexandra Fjord Highlands at relative abundances <0.14% (Supplementary 8 and 10). Light-harvesting complex I (*LHCA1-5*) and II (*LHCB1-7*) and chlorophyll a/b binding light-harvesting proteins (*pcbA-H*) were not detected in any of the MAGs (Supplementary 10). Genes for the protochlorophyllide complex subunits, *bchN* and *bchB*, were found in MAGs of the *Rubrobacteraceae* family of *Actinobacteriota*; but in members of *Rubrobacteraceae* these genes do not appear to be connected to bacteriochlorophyll-based phototrophy [58, 59]. MAGs assigned to the genus *Amaricoccus* (*Rhodobacteraceae*, *Alphaproteobacteria*) contained *pufL*, *pufM* and *bchB* genes although RuBisCO genes were undetected (Supplementary 8 and 10). Proteorhodopsin genes with DTE motifs in the 3rd transmembrane helix were identified in our *Bacteroidota* and *Deinococcota* MAGs, indicating the potential for photoheterotrophy [60] (Supplementary 14). Although photosynthetic markers are limited throughout the MAGs and metagenomes, suggesting a low capacity for photosynthesis, their presence could still result in significant expression and activity.

## Trace gas oxidation supports maintenance and productivity in arid and hyperarid ecosystems across the poles

Functional gene analysis suggests that phototrophy and the oxidation of geochemical compounds have a limited capacity to support the electron transport chain and drive carbon fixation within the polar desert microbiomes studied here. Conversely, there was an extensive genetic capacity for trace gas oxidation, with high-affinity [NiFe]-hydrogenases from group 1h, 1l and 2a distributed across all 18 soil metagenomes (Fig. 2 and Supplementary 9), with either groups 1h or 1l detected in a third of all retrieved MAGs (≥50% completeness, ≤10% contamination) (Fig. 3 and Supplementary 15). Phylogenetic analysis revealed a novel clade of [NiFe]-hydrogenases (Supplementary 16), hereby referred to as group 1m. Like groups 1h and 1l [NiFe]-hydrogenase, group 1m was detected in all metagenomes (Supplementary 9) and a further 13 of the obtained MAGs, all of which are members of *Actinobacteriota* (Supplementary 15). Group 1l [NiFe]-hydrogenase was identified in the phyla *Deinococcota* (order *Deinococcales*) (Bin#: 160, 161) with widespread presence of this novel hydrogenase also detected within *Actinobacteriota*, *Bacteroidota*, *Chloroflexota* and *Proteobacteria* [24] (Supplementary 15). MAGs containing the well-established high-affinity group 1h [NiFe]-hydrogenases were widely distributed in seven previously established trace gas oxidizing phyla: *Acidobacteriota*, *Actinobacteriota*, *Chloroflexota*, *Ca*. Dormibacterota, *Ca*. Eremiobacterota, *Proteobacteria* and *Verrucomicrobiota* [11, 22, 61, 62] (Figs. 3 and 4 and Supplementary 15).

Analysis of the arrangement of genes within the 1m [NiFe]-hydrogenase gene cluster shows structural similarities to the well-characterized high-affinity 1h [NiFe]-hydrogenase, as well as the recently discovered high-affinity 1l [NiFe]-hydrogenase, implying that 1m [NiFe]-hydrogenases is also a high-affinity enzyme. Simultaneously, key differences that justify classification of these sequences into a novel grouping were consistently observed. For example, as found for the 1h [NiFe]-hydrogenase gene cluster, the small and large subunits of 1m [NiFe]-hydrogenase were encoded by adjacent genes. This contrasts with the form 1l [NiFe]-hydrogenase gene cluster which, consistent with a previous study [24], has five short predicted transmembrane proteins (HylTM1-5) interposing the large and small subunits. The group 1m [NiFe]-hydrogenase gene cluster typically also contained the following genes, most of which are unique to this novel group: HybD peptidase involved in processing of the hydrogenase large subunit [63]; an FeS cluster assembly protein; a Ni insertion ATPase/GTPase (CooC-type); tetratricopeptide repeat protein (in general involved in assembly of multiprotein complexes); a bifunctional ligase/repressor (BirA) homolog; a DUF1059 domain (of unknown function); and two small proteins (82–92 amino acids) with no identifiable domains that each contain a single transmembrane helix (Supplementary 17). The group 1h [NiFe]-hydrogenase from *Cupriavidus nectator* H16 (PDB ID = 5AA5) [64] was consistently identified as the best model for the 1m [NiFe]-hydrogenase amino acid sequences extracted from our MAGs (Phyre2 [65]; *hhmL* 87–89% residues modeled at >90% confidence, *hhmS* 68–79% residues modeled at >90% confidence; SWISS-MODEL [66] Global Model Quality Estimate = 0.67–0.72 for the whole tetramer). From this informatic analysis, we conclude that 1m [NiFe]-hydrogenase is an evolutionarily and structurally distinct group of high-affinity enzymes. Enzyme purification and characterization studies, including X-ray crystallography and nuclear magnetic resonance spectroscopy [67, 68], are needed to verify this identification biochemically.

In terms of CO oxidation, proteins annotated as the aerobic carbon monoxide dehydrogenase large subunit (*coxL*) were highly prevalent across the 230 dereplicated MAGs. However, as most of these protein sequences lacked the catalytic cluster of CODH [69], we infer that only 18 of the 230 MAGs contained genes for actual CODH. These included members of the *Actinobacteriota*, *Chloroflexota* and *Ca*. Dormibacterota (Supplementary 15).

RuBisCO form IE accounted for 68% of all RuBisCO genes identified in the metagenomes, dominating in samples from the Antarctic sites; Mitchell Peninsula, The Ridge and New Harbour (average relative abundances of 18%, 17% and 8.1%, respectively), and occurring in lower abundances in Tibetan Plateau, Spitsbergen Svalbard and Alexandra Fjord Highlands samples (3.1%, 1.9% and 1.4%, respectively; Fig. 2 and Supplementary 9). Although widely distributed, the photosynthetic RuBisCO forms IA and 1B were in low abundance throughout all metagenomes (average 1.1% and 0.2%, respectively), particularly in comparison to RuBisCO form IE.

RuBisCO form IE was encoded in 38 of the obtained MAGs, while RuBisCO forms II, III, IA, IB, IC and ID were collectively limited to only five MAGs (Fig. 3 and Supplementary 15). Overall, 25 MAGs contained both a detectable RuBisCO form IE and high-affinity hydrogenase genes (Fig. 4 and Supplementary 15). Of these, 18 belonged to the phylum *Actinobacteriota*, three to the phylum *Chloroflexota* (family *Ktedonobacteraceae* including novel genera UBA11361 and CF-113) and two each to the proposed trace gas chemosynthetic phyla *Ca*. Dormibacterota and *Ca*. Eremiobacterota [33, 34]. Of these 25 MAGs, eight also encoded CODH with an active-site loop [69], The eight MAGs containing all three genes (*rbcL1E*, *hhyL*/*hylL*/*hhmL* and CODH) belong to the *Actinobacteriota* (order *Mycobacteriales* including novel genus QHCD01, and order *Solirubrobacterales* including novel genus Palsa-465), *Chloroflexota* (two novel genera in the family *Ktedonobacteraceae*, CF-113 and UBA11361) and *Ca*. Dormibacterota (genus *Candidatus* Dormibacter) (Fig. 4 and Supplementary 15). Therefore, these taxa potentially possess high metabolic flexibility, with the potential to use both H_2_ and CO for hydrogenotrophic and carboxydotrophic growth, respectively (Fig. 4 and Supplementary 10 and 15).

Only a single MAG (classified as *Actinobacteriota*, family *Solirubrobacteraceae*) encoded both CODH and RuBisCO form IE but no detectable high-affinity hydrogenase gene (Supplementary 15). This suggests that in cold desert microorganisms, CO oxidation is rarely the sole driver of atmospheric chemosynthesis, occurring most frequently in conjunction with H_2_ oxidation.

## Atmospheric chemosynthesis and photosynthesis co-occur in microbial communities to support primary production

**Fig. 5:**
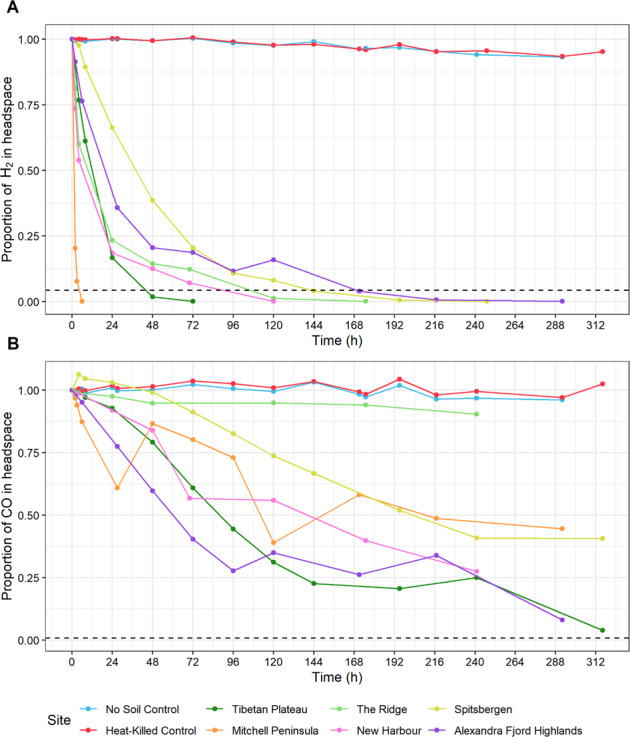
The oxidation of atmospheric gases by surface soil microcosms from six cold deserts. The oxidation of **A** hydrogen and **B** carbon monoxide are displayed. Values shown have been normalized against the starting concentration of each gas and are the mean of biological triplicates. The dashed line in **A** indicates atmospheric H_2_ (530 p.p.b.v) and in **B** indicates atmospheric CO (90 p.p.b.v). Rapid high-affinity hydrogenase activity was observed across all sites (Average H_2_ consumption; Mitchell Peninsula 421.4 nmol/mol/h/g, NH 42.6 nmol/mol/h/g, The Ridge 41.1 nmol/mol/h/g, TP 35.6 nmol/mol/h/g, Alexandra Fjord Highlands 21.6 nmol/mol/h/g, SS 9.4 nmol/mol/h/g), particularly within the Mitchell Peninsula microcosms, each of which consumed hydrogen to sub-atmospheric levels within 6 h of incubation. Carbon monoxide consumption was observed however, compared to hydrogen consumption, these rates were slower and varied greatly between samples within each site.

Gas chromatography was used to confirm scavenging and oxidation of atmospheric H_2_ and CO in soil microcosms from all six global desert sites. Headspace H_2_ concentrations rapidly dropped to sub-atmospheric levels (Fig. 5A and Supplementary 18), with average atmospheric hydrogen oxidation rates ranging from 9.4 nmol/mol/h/g at The Ridge through to 421.4 nmol/mol/h/g at Mitchell Peninsula. Antarctic and Tibetan Plateau soil microcosms demonstrated the highest H_2_ oxidation rates, consistent with the higher abundances of *Actinobacteriota* within these samples (Fig. 1 and Supplementary 5, 6 and 18). Mitchell Peninsula microcosms demonstrated extremely rapid H_2_ oxidation, likely reflecting the high abundances of the phyla *Ca*. Eremiobacterota and *Ca*. Dormibacterota (average 7.8% and 3.6%, respectively) that are proposed to be capable of atmospheric chemosynthesis (Fig. 1 and Supplementary 5, 6 and 18). The rapid H_2_ uptake rates reported here far exceed those previously calculated to be required to sustain the energy needs of similarly structured polar and temperate terrestrial microbiomes [11, 22, 24, 37, 70–72], such as those from Robinson Ridge (3.49 nmol/mol/h/g) and Adams Flat (5.54/nmol/mol/h/g) soils in Eastern Antarctica [11], as well as cultured bacterial isolates [19].

CO oxidation was also observed in soil microcosms from all desert sites except The Ridge (Fig. 5B). However, the rates observed were much slower than for H_2_ oxidation in the same microcosms, and high levels of variation in CO oxidation rates were observed between soils from the same site (Supplementary 18). These results, combined with the greater abundance of putative H_2_ oxidizing bacteria compared to putative CO oxidizers in the soil samples, suggests that atmospheric H_2_ oxidation is a more important and widespread energy acquisition process (c.f., CO oxidation) in these polar soil microbiomes.

**Fig. 6:**
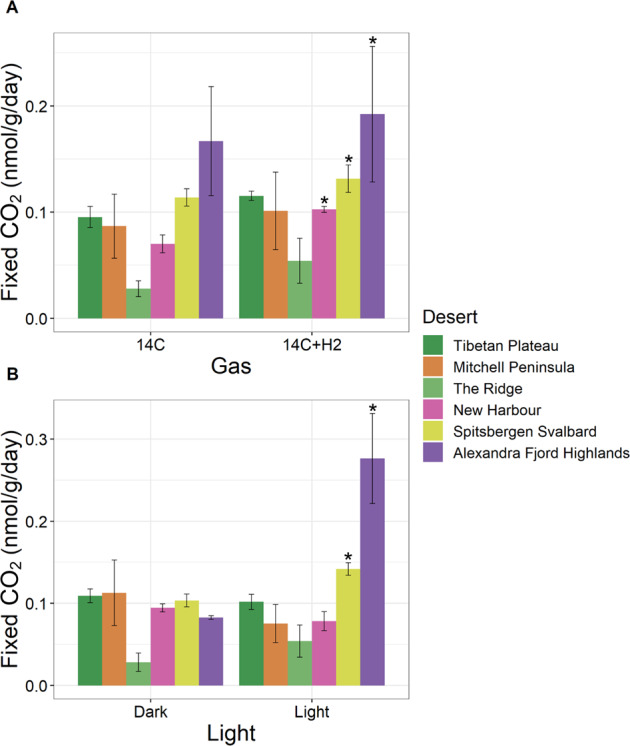
Changes in ^14^CO_2_ assimilation in soils from six cold deserts under differing abiotic conditions. Increases in carbon assimilation under **A** hydrogen stimulation and **B** light exposure are displayed. Carbon assimilation by soil microcosms from all deserts were consistently stimulated by the addition of atmospherically relevant hydrogen concentrations (~10 p.p.m.v), with significant increases observed within microcosms from New Harbour (*p* = 0.013), Spitsbergen Svalbard (*p* = 0.033) and Alexandra Fjord Highlands (*p* = 0.031). Light exposure also led to a significant increase in carbon assimilation within soils from Spitsbergen Svalbard and Alexandra Fjord Highlands (*p* = 0.022 and 0.015, respectively), but did not significantly influence primary production across the other deserts. Normality was determined using Shapiro–Wilk tests. When the data were normally distributed, statistical significance was determined using a two-tailed paired *t-*test. Otherwise, a two-tailed Wilcoxon signed-rank test with a Bonferroni correction was implemented.

We also demonstrated that atmospheric chemosynthesis contributes to primary production in globally-distributed cold desert soils (Fig. 6). Whilst Ji et al. [11] investigated atmospheric chemosynthesis in two Eastern Antarctic sites, significant increases (*p* < 0.05) in carbon fixation under hydrogen stimulation were only observed in soils obtained from one of these sites, Adams Flat [11]. Furthermore, in this previous study, the average TC assimilation per sample across all conditions was 31.6 pmol at Adams Flat and 7.1 pmol at Robinson Ridge [11], which is comparable to the values reported here (Alexandra Fjord Highlands: 49.2 pmol, SS: 15.7 pmol, Mitchell Peninsula: 14.5 pmol, TP: 11.5 pmol, NH: 7.9 pmol, The Ridge: 3.1 pmol), strengthening the conclusion that atmospheric chemosynthesis is a significant contributor to the global carbon flux. Here, ^14^CO_2_ assimilation was consistently higher in New Harbour (*p* = 0.013), Alexandra Fjord Highlands (*p* = 0.031) and Spitsbergen Svalbard (*p* = 0.033) soil microcosms with H_2_ supplementation compared to those without H_2_ addition. In the high Arctic desert soils, light exposure also led to a significant increase (*p* = 0.015 and *p* = 0.022, respectively) in carbon fixation, highlighting the co-occurrence of atmospheric chemosynthesis and photosynthesis. Genes encoding elements of the photosynthetic apparatus were detected in the metagenomes (Fig. 3 and Supplementary 9), with higher rates of photosynthesis within the high Arctic sites likely attributed to a higher abundance of photosynthetic taxa, particularly *Cyanobacteria* in the Alexandra Fjord Highlands (Fig. 1 and Supplementary 5). Consistent with previous studies [73–75], greater edaphic *Cyanobacteria* abundances may be a reflection of greater moisture availability at these sites (0.077% Alexandra Fjord Highlands, 0.243% SS) [12]. Furthermore, this may in turn account for a lower relative abundance of chemoautotrophic and desiccation-tolerant taxa, particularly *Actinobacteria*, within the Arctic microbiomes [5, 75]. Conversely, light exposure did not stimulate significant ^14^CO_2_ fixation rates in Mitchell Peninsula soils, although both variabilities in ^14^CO_2_ assimilation rates and bacterial community structure were observed for the three Mitchell Peninsula soils examined. For example, MP3 was comprised of a higher abundance of photosynthetic *Cyanobacteria* (1.1% relative abundance), with higher levels of ^14^CO_2_ fixation in the presence of light observed in contrast to MP1 and MP2 soils (0.2% and 0.4% relative *Cyanobacteria* abundance, respectively) (Fig. 1 and Supplementary 5). It should be noted that in MP3 higher *Cyanobacteria* abundances co-occurred with higher relative abundances of trace gas chemosynthetic phyla *Ca*. Eremiobacterota (5.6%) and *Ca*. Dormibacterota (12.3%). In MP2, a low abundance of photosynthesis genes combined with a high relative abundance of *Ca*. Eremiobacterota (5.1%) and *Ca*. Dormibacterota (10.9%) was found, whilst genetic markers for photosynthesis and atmospheric chemosynthesis were both low in MP1 (0.2% *Ca*. Eremiobacterota) and (0.3% *Ca*. Dormibacterota). With such great taxonomic variation between biological replicates, it is unsurprising that variable biochemical activity was also observed at this site (Supplementary 18).

## RuBisCO form 1E phylogeny spans eight bacterial phyla inhabiting environmental reservoirs

To complement the diversity of atmospheric chemosynthesis genetic determinants uncovered in our MAGs, we extracted a further putative 4507 RuBisCOs, 1073 high-affinity hydrogenases and 1289 aerobic CODH based on sequence identity (>30% to representative sequences, 70% alignment) from 24,080 bacterial and archaeal representative genomes from release R04-R89 of the GTDB. Phylogenetic analyses confirmed the identity of the extracted RuBisCO sequences (190 form IE, 291 form ID, 251 form IC, 194 form IB, 423 form IA, 348 form II, 275 form III and 1188 form IV) (Supplementary 19) and high-affinity hydrogenase groups (722 form 1h, 6 form 1m, 19 form 1l) (Supplementary 16). Consistent with analysis of our own MAGs, RuBisCO form IE was widely distributed, found within *Actinobacteriota*, *Chloroflexota*, *Ca*. Dormibacterota and *Ca*. Eremiobacterota (Fig. 4 and Supplementary 15) and, in accordance with previous studies [11, 27], *Firmicutes* and *Verrucomicrobiota* (Fig. 4). High-affinity group 1h [NiFe]-hydrogenases co-occurred with RuBisCO form IE within MAGs from each of these six trace gas oxidizing phyla detected, whilst CODH co-occurred with RuBisCO form IE in all phyla except *Verrucomicrobiota* (Fig. 4 and Supplementary 15). Through this analysis, we also discovered RuBisCO form IE within *Acidobacteriota* (*n* = 5) and *Deinococcota* (*n* = 14) genomes. Although the co-occurrence of high-affinity hydrogenases were not detected within these genomes, four of the *Deinococcota* genomes encoded CODH (Fig. 4), suggesting a capacity for atmospheric chemosynthesis using carboxydotrophy rather than hydrogenotrophy (Fig. 4). Therefore, in addition to *Actinobacteriota*, *Ca*. Dormibacterota and *Ca*. Eremiobacterota, the bacterial phyla *Chloroflexota*, *Deinococcota*, *Firmicutes* and *Verrucomicrobiota* are now implicated as being capable of atmospheric chemosynthesis through H_2_ and/or CO oxidation strategies.

Of the GTDB genomes that contained both RuBisCO form IE and group 1h [NiFe]-hydrogenase genes, 80 were obtained from the analysis of pure cultured microorganisms, rather than MAGs. *Actinobacteriota* accounted for most of these isolates (*n* = 76) (orders *Mycobacteriales*, *Streptomycetales*, *Streptosporangiales* and *Solirubrobacterales*), with *Firmicutes* (*Sulfobacillus thermosulfidooxidans*), *Chloroflexota* (*Nitrolancea hollandica*) and *Verrucomicobiota* (*Methylacidiphilum kamchatkense*) also represented. Of these, the *Firmicutes* and 35 of the 76 *Actinobacteriota* genomes also encoded CODH. In addition to the 80 isolates described, a further 12 of the genomes obtained from microbial isolates contained RuBisCO form IE and CODH but did not have a detectable group 1 [NiFe]-hydrogenase gene. This provides further indication that a subset of taxa capable of atmospheric chemosynthesis are likely to utilize carboxydotrophic rather than hydrogenotrophic growth strategies. Like the isolates that demonstrated both carboxydotrophic and hydrogenotrophic markers, these taxa also spanned *Actinobacteriota* and *Firmicutes*, but included a third phylum, *Deinococcota* (*Meiothermus cerbereus*). The discovery of atmospheric chemosynthetic genes within already isolated microorganisms opens the possibility for more extensive culture-dependent research that could conclusively characterize the activation conditions and mechanisms of the underlying atmospheric chemosynthetic pathways.

Overall, 92 GTDB reference genomes obtained from bacterial isolates are inferred to have the capacity for atmospheric chemosynthesis by encoding RuBisCO form IE and either aerobic CODH or group 1h [NiFe]-hydrogenase or both. These bacteria have been previously isolated from a broad range of temperate and desert environments, the majority from soil. Soil environments included agricultural fields [76, 77], karst caves [78, 79], plant matter [80, 81] and mining and ore deposits [82]. These putative trace gas chemosynthetic bacteria were also isolated from aqueous environments, including ocean [83], sediment [84–86], lakes [87], river and stream [88, 89], hot spring [90, 91] and groundwater samples [92]. Aqueous environments such as these generally have a lower capacity for oxygenation compared to terrestrial systems, highlighting the need to confirm activation of the chemoautotrophic pathways in these organisms and characterize the underlying metabolic capacities. Ultimately, this diverse habitat range supports the hypothesis that high-affinity H_2_ and CO oxidation is a globally pervasive mechanism of energy acquisition that supports microbial survival, and potentially cellular growth, in a wide array of microbial taxa.

## Conclusion

Atmospheric chemosynthesis supplements photosynthetic primary production in cold desert soils across the globe, with trace gas oxidization providing the energy and/or carbon needs to sustain terrestrial ecosystems in the high Arctic, Antarctica and Tibetan Plateau. This observation expands the significance of soil microorganisms as key elements in the global carbon budget. We have informatically identified a novel high-affinity hydrogenase, termed 1m [NiFe]-hydrogenase, and increased the list of potential trace gas chemosynthetic phyla to seven, with key enzymes co-occurring within MAGs from four previously unidentified bacterial phyla: *Chloroflexota*, *Firmicutes*, *Deinococcota* and *Verrucomicrobiota*. The discovery of a suite of putative trace gas chemosynthetic bacteria from diverse habitats, and their previous isolation under nutrient-rich conditions, highlights their proposed metabolic flexibility- being capable of growth and persistence through a combination of heterotrophic, carboxydotrophic and hydrogenotrophic strategies. Finally, the isolation of presumptive trace gas chemotrophs from a range of environmental reservoirs provides new opportunities to experimentally demonstrate and confirm the underlying metabolic pathways of atmospheric chemosynthesis and to clarify the physiological importance of the energy generation and carbon fixation processes in bacterial survival.

## Materials and methods

### Soil sampling

Two Eastern Antarctic sites were chosen for inclusion in this study: Mitchell Peninsula (66°31´S, 110°59´E) from the Windmill Islands region and The Ridge (68°54’S, 78°07’E) from the Vestfold Hills region. New Harbour (NH) (77°34’S, 163°31’E), lower Taylor Valley (McMurdo Dry Valleys) was also investigated, as were two high Arctic sites; Alexandra Fjord Highlands (78°51’N, 75°54’W) in Canada and Spitsbergen Svalbard (SS) (78°14’N, 15°25’W) in Norway. Samples were also collected from the cold, high-altitude Qinghai-Tibet Plateau (TP) (32˚ 27’N, 80˚4’E) in Western China. Sampling of Mitchell Peninsula, The Ridge, Alexandra Fjord Highlands and SS was conducted under auspices of the Australian Antarctic Program. NH was sampled by the Centre for Microbial Ecology and Genomics, University of Pretoria, and the TP soils were obtained from the Institute of Tibetan Plateau Research, Chinese Academy of Sciences. Mitchell Peninsula, Alexandra Fjord Highlands and SS were sampled between 2005 and 2008, The Ridge was sampled in 2012, TP was sampled in 2015 and NH was sampled in 2018. During sampling, three 50 x g surface soils (<10 cm) were aseptically collected from each of these sites and sieved to <2 mm (total samples; 18) [93, 94]. TP samples were obtained from a randomly selected 1 × 1 m quadrant. For all other sites, samples were obtained from 100 m intervals along a spatially explicit transect that is 300 m long and 3 m wide (Siciliano et al. [93]). All soil samples were stored at −80 °C until used in this study.

### Physicochemical analysis

Physicochemical data describing the Mitchell Peninsula, The Ridge, SS, Alexandra Fjord Highlands and The Ridge soils was obtained from previous publications [12, 29–31]. For the Antarctic and Arctic sites, these values were originally derived from data obtained from the Biome of Australia Soil Environments repository (https://data.bioplatforms.com/organization/about/australian-microbiome) [95] and the Australian Antarctic Datacentre (10.4225/15/526F42ADA05B1). Physicochemical data describing the NH soils was conducted during this study. This data was obtained using standard procedures described previously [93, 96]. In brief, total carbon (TC) was measured using combustion and nondispersive infrared analysis, total organic carbon was measured using the Walkley-Black chromic acid wet oxidation method [97], and pH was measured using a 1:5 soil to distilled water suspension [98]. Dry matter fraction was measured by comparing the weight of ~10 g soil prior to and after drying in an oven at 105 °C for 55 h.

### Community DNA extraction, sequencing and cell abundance estimations

DNA was extracted in triplicate from 0.25–0.30 g of each soil sample using the FastDNA SPIN kit for soil (Mitchell Peninsula Biomedicals, NSW, Australia) as per the manufacturer’s instructions. Metagenomic shotgun libraries were prepared from DNA extractions using the Nextera XT DNA Sample Preparation Kit (Illumina). Sequencing was performed on an NextSeq500 platform (Illumina) with 2 × 150 base pair high output run chemistry and 7 Gb coverage per sample. The cell abundance within each soil sample was estimated using quantitative polymerase chain reaction targeting the 16S rRNA gene, according to previously described methods [12].

### Metagenome assembly and binning

Low quality reads were identified and removed with Trimmomatic [99] using a sliding window of 4 bases with an average quality of 21 (SLIDINGWINDOW:4:21), with those reads less than 50 bp in length discarded (MINLEN:50). Quality controlled reads were then assembled using MEGAHIT (ver. 1.2.2-beta) [100] with default parameters. Contig statistics including assembly size, number of contigs, contig length distribution, and N50/90 values were calculated with BBMap (ver. 38.41) [101] and custom scripts. Quality controlled reads for each sample were mapped onto their respective assemblies with minimap2 as part of CoverM “make” (ver. 0.3.0, B. Woodcroft, unpublished, https://github.com/wwood/CoverM). Low quality mappings were removed with CoverM “filter” (minimum identity 95% and minimum aligned length of 75 bp). To estimate the coverage of the metagenomes, Nonpareil [102] was run using the quality controlled reads, K-mer alignment method and default parameters. Assemblies for each sample were binned by providing the contigs for each sample and filtered BAM files as input to UniteM (ver. 0.0.15; D. parks, unpublished, https://github.com/dparks1134/UniteM) and using a minimum contig length of 1500 bp and Maxbin (ver. 2.2.4) [103], MetaBAT (ver. 0.32.5) [104] and MetaBAT2 (ver. 2.12.1) [105] binning methods (max40, max107, mb2, mb_verysensitive, mb_sensitive, mb_specific, mb_veryspecific and mb_superspecific).

Bin completeness and contamination was evaluated using CheckM (ver. 1.0.12) [106] and the taxonomy assigned using the Genome Taxonomy Database Toolkit (GTDB-Tk; ver. 1.3.0; with reference to GTDB R05-RS95) [107]. Binning yielded 860 bins (17 archaeal and 811 bacterial), 282 of which were ≥50% complete with ≤10% contamination. A non-redundant set of bins were obtained by dereplicating with dRep (ver. 2.2.3, sa = 0.95) [108]. Following dereplication, 230 metagenome-assembled genome (MAG) bins (7 archaeal and 223 bacterial; ≥50% completeness, ≤10% contamination) were selected for further analysis. Of the 230 MAGs, 76 were estimated to be more than 90% complete and <5% contaminated (Supplementary 8).

### Calculation of MAG abundances

To calculate the relative abundance of each MAG, reads from each sample were mapped to the set of MAGs using CoverM “make”. Low quality mappings were removed with CoverM “filter” (minimum identity 95% and minimum aligned length of 75 bp). The mean coverage of each MAG was calculated with CoverM, with those with a fraction of coverage <5% reported as having zero coverage. The relative abundance of each MAG, among those obtained, was calculated as its coverage divided by the total summed coverage of all MAGs. Abundance values were multiplied by the fraction of reads that mapped to all MAGs to produce the relative abundance of each MAG within the entire sample.

### Custom database generation and metabolic annotation of metagenomic short reads

The abundance of 43 genes, including the different forms of RuBisCO (IA, IB, IC, ID, IE, II, III and IV) and high-affinity [NiFe]-hydrogenase (1h, 1l, 1m and 2a), were determined for each metagenome. Custom databases for each gene were first generated by downloading representative (seed) sequences for RuBisCO_large from Pfam [109] (PF00016) and group 1h [NiFe]-hydrogenase large subunit from the HydDB [62]. Potential RuBisCO and hydrogenase sequences were then identified in representative genomes from the GTDB (R04-RS89) by first predicting the gene protein-coding sequences (CDS) in each genome using Prodigal (ver. 2.6.3) as part of Prokka (ver. 1.12) and then aligning the resulting protein sequences against the representative RuBisCO_large and group 1h [NiFe]-hydrogenase large subunit sequences with BLAST+ (ver. 2.9.0; -max_hsps 1) [110]. BLAST results were filtered to those hits where at least 30% sequence identity and 70% alignment of the representative sequence was achieved, and false positives manually removed. Separate phylogenetic analysis was performed upon the filtered RuBisCO and hydrogenase sequences alongside representative sequences. RuBisCO subtypes were identified according to the clades formed and compiled into separate protein databases. Hydrogenase sequences that clustered with the group 1h, group 1l, group 1m and group 2a [NiFe]-hydrogenases clade (Supplementary 16) were also compiled into separate protein databases. An additional 35 protein databases were generated using sequences retrieved from UniprotKB protein database (March 2020) [111] (Supplementary 20). These protein sequences included those associated with the CBB cycle, energy metabolism, nitrogen cycling, photosynthesis, the rTCA cycle and trace gas oxidation.

Gene abundances were then calculated by aligning reads against each of the 43 reference protein databases using DIAMOND BLASTX [112], with a query coverage of 80% and an identity threshold of 50%. To account for differences in sample sequencing depth and gene length, read counts were normalized to reads per kilobase per million (RPKM) and further normalized against the mean RPKM value estimated from 14 single-copy ribosomal marker genes derived from SingleM (ver. 0.13.0; unpublished, https://github.com/wwood/singlem) and phylosift [113] to infer the percentage of the community encoding the gene [24]. These results are provided in Supplementary 9 and were also visualized as a heatmap using the R package ggplot2 [114] (Fig. 2).

To further assess the functional capacities of the metagenomes, a non-redundant gene catalog for all samples was constructed by first predicting protein-coding sequences (CDS) in the assembled scaffolds using Prodigal (ver. 2.6.3) in metagenomic mode, with those complete sequences, including start and stop codons, extracted using mfqe (ver. 0.5.0; B. Woodcroft, unpublished, https://github.com/wwood/mfqe). Protein sequences were clustered at 100% protein identity using CD-HIT (ver. 4.8.1) [115], with all members of each cluster required to have at least 80% of their sequence overlapping with the longest (seed) sequence. Sequences were then annotated using DRAM (ver. 1.3.3) [116].

### Community taxonomic profiling of the metagenomes

The taxonomic profile of each unassembled metagenome was determined through the classification of reads corresponding to the universal single-copy ribosomal marker protein L16/L10E *rplP* (Lan et al., [117]) using the approach described by Ortiz et al. [24]. Briefly, *rplP* sequences for representative bacterial and archaeal genomes in the GTDB R05-RS95 were downloaded (https://data.ace.uq.edu.au/public/gtdb/data/releases/release95/95.0/). GraftM (ver. 0.12.2) [118] was then used to create a gene family specific phylogenetic package, which was used to create a classification package for SingleM (ver. 0.13.2). An operational taxonomic unit (OTU) profile was then generated using SingleM “pipe” on the paired reads for each sample. This taxonomic profile was visualized as a bar chart at the phyla-level using the R package ggplot2 [114] (Fig. 1). Rarefaction analysis was performed by sub-sampling the reads for all samples in increments of 1,000,000 read pairs, up to the lowest total read pair count among the samples (~27,000,000). SingleM was then run on each increment to generate a community profile based upon the coverage of OTUs for the *rpIP* gene. The number of genera within each profile was plotted, excluding OTUs not classified to a genus level. To complement the marker gene profiling, metagenomes were also taxonomically profiled by mapping paired reads against the representative GTDB genomes with minimap2 [119] as part of CoverM “make” (ver. 0.4.0) (Supplementary 6). Low quality mappings were removed with CoverM “filter” (minimum identity 95% and minimum aligned length of 75%). Forward reads that remained unmapped or were filtered by CoverM were profiled by Kaiju (ver. 1.7.3) using the *nr* + euk database [120]. All taxonomic profiles constructed are described at a class level (Supplementary 5–7).

### Gene extraction and functional annotation of MAGs

MAGs were translated and functionally annotated using a combination of Prokka (ver. 1.14), Prodigal (ver. 2.6.3), the carbohydrate-active enzymes database (CAZy) [121], NCycDB [122] and EnrichM (ver. 0.4.15, J. Boyd, unpublished, https://github.com/geronimp/enrichM), the latter using annotation options --ko_hmm, --pfam, --tigrfam, --orthologs, --clusters, --cazy and --ec. In EnrichM, for a query gene to be considered for annotation, the minimum fraction aligning to a reference, and vice versa, was set to 0.5, with a minimum percent identity of 30% also required. Sequences initially annotated as CODH large subunit (*coxL*) were manually inspected for the presence of the CODH active-site loop [69] to determine if they were likely to be *coxL*; using this approach, the majority of *coxL* homologs were removed from further consideration. Rhodopsin amino acid sequences were identified through annotation against the Pfam [109] database using InterProScan [123], and TMHMM [124] used to subsequently confirm the presence of seven transmembrane helices. Rhodopsin sequences were then aligned with MAFFT (ver. 7.407) [125, 126] and visualized with Geneious Prime 2021.0.1 (https://www.geneious.com). Rhodopsin motifs were identified in the 3rd transmembrane helix while the retinal binding motifs were identified in the 7th transmembrane helix.

### Phylogenetic analysis of RuBisCO and hydrogenase within MAGs and GTDB genomes

Potential RuBisCO and high-affinity group 1 [NiFe]-hydrogenase large subunit sequences were identified in the 860 MAGs obtained in this study and 24,080 representative genomes from the GTDB R04-RS89 using BLAST+ (ver. 2.9.0; -max_hsps 1) [110]. BLAST results were filtered to those hits where at least 30% sequence identity and 70% alignment of the representative sequence was achieved, and false positives manually removed. Phylogenetic analysis was conducted to identify the subtype of each RuBisCO and hydrogenase sequence extracted (Fig. 4 and Supplementary 16 and 19). In total, 4622 putative RuBisCO sequences were obtained: 115 from our constructed MAGs and 4507 from representative genomes from the GTDB. In addition, 1377 putative hydrogenase sequences were obtained: 304 from our MAGs and 1073 from representative genomes. Identical sequences were removed from further analysis.

Separate phylogenetic analyses were performed upon the extracted RuBisCO and hydrogenase sequences. Multiple sequence alignment was performed using MAFFT (ver.7.407), employing the L-INS-i iterative refinement method [125, 126]. The resulting alignments were then trimmed to remove poorly aligned regions using trimAl (ver. 1.4.1), with a gap threshold of 0.5 [127]. Sequences with more than 50% gaps after alignment were removed. Maximum likelihood phylogenetic trees were constructed using IQ-Tree (ver. 1.6.10) [128], applying 1000 ultrafast bootstrap iterations, hill-climbing nearest neighbor interchange search and incorporating additional SH-like approximate likelihood ratio tests (SH-alrt) [129]. ModelFinder was performed to determine the best phylogenetic model, which was the amino-acid exchange rate general matrix (LG) plus “FreeRate” model heterogeneity (+R9 for the RuBisCO tree and +R10 for the hydrogenase tree) [128]. Sequences that failed the chi-square test during tree building were removed. The final consensus trees comprised 3255 RuBisCO sequences and 2103 hydrogenase sequences and were both uploaded to iTOL [130] for visualization. Branches within the hydrogenase tree were color-coded according to the form of hydrogenase and bootstrap values 90–100 indicated by circles on the corresponding branches. Within the RuBisCO tree, sequences were color-coded by phyla and branches color-coded by RuBisCO form. MAGs were color-coded according to the cold-desert site where they were primarily detected. Representative genomes and MAGs harboring high-affinity hydrogenase and aerobic carbon monoxide dehydrogenase sequences were marked with triangles. A complete tree depicting all 3255 RuBisCO sequences is provided (Supplementary 19), as is a pruned version focussing upon the entire RuBisCO form IE clade, the 115 RuBisCO sequences extracted from the cold desert MAGs and their closest corresponding references sequences within genomes from the GTDB (Fig. 4). Copy numbers obtained for the MAGs were converted to presence/absence, and the proportion of MAGs from each phyla containing the genes visualized as a heatmap using the R package ggplot2 [114] (Fig. 3).

### Gene structural analysis and homology modeling of 1m [NiFe]-hydrogenase

Amino acid sequences encoding the 1m [NiFe]-hydrogenase small subunit (*hhmS*) and large subunits (*hhmL*), as well as the surrounding genes were extracted from four of the MAGs assembled in this study (bin #: 90, 63, 45, 35). All sequences were submitted to ExPASy BLAST (using the “UniProtKB/Swiss-Prot only” option) [131] and to InterProScan [132] to identify functional domains, and potential subcellular location (e.g., transmembrane helices). Amino acid sequences encoding the 1m [NiFe]-hydrogenase small and large subunits from all the 4 MAGs were also modeled through input into the Protein Homology/analogY Recognition Engine V 2.0 (Phyre2) using the intensive modeling mode [65]. The amino acid sequence of the group 1m [NiFe]-hydrogenase large and small subunits (*hhmSL*) from each of the four MAGs were input into SWISS-Model [66], to visualize the structure of the group 1m [NiFe]-hydrogenase tetramer.

### H_2_ oxidation and ^14^CO_2_ fixation assays

Gas chromatography was used to measure the activity of high-affinity hydrogenases and carbon monoxide dehydrogenases within microbial communities from each cold desert region. To determine whether the microbial communities within each soil sample were fixing carbon through atmospheric chemosynthesis, photosynthesis, a combination of both or neither, ^14^CO_2_ assimilation assays were conducted. Both assays and the subsequent statistical analysis of the results were conducted according to previously described methods [11] (Supplementary 21).

## Supplementary information

Supplementary 4

Supplementary 13

Supplementary 14

Supplementary 16

Supplementary 17

Supplementary 18

Supplementary 19

Supplementary 21

Supplementary 1

Supplementary 2

Supplementary 3

Supplementary 5

Supplementary 6

Supplementary 7

Supplementary 8

Supplementary 9

Supplementary 10

Supplementary 11

Supplementary 12

Supplementary 15

Supplementary 20

## Data Availability

Next generation sequencing data that supports the findings of this study have been deposited in GenBank with the accession code PRJNA664610. All other data supporting the findings of this study are available in the article/Supplementary Information.
