# Supplementary figures and images for: Atmospheric chemosynthesis is phylogenetically and geographically widespread and contributes significantly to carbon fixation throughout cold deserts
Source: ISME J. 2022 Aug 6;16(11):2547–60. doi: 10.1038/s41396-022-01298-5 (PMC9561532; doi:10.1038/s41396-022-01298-5)

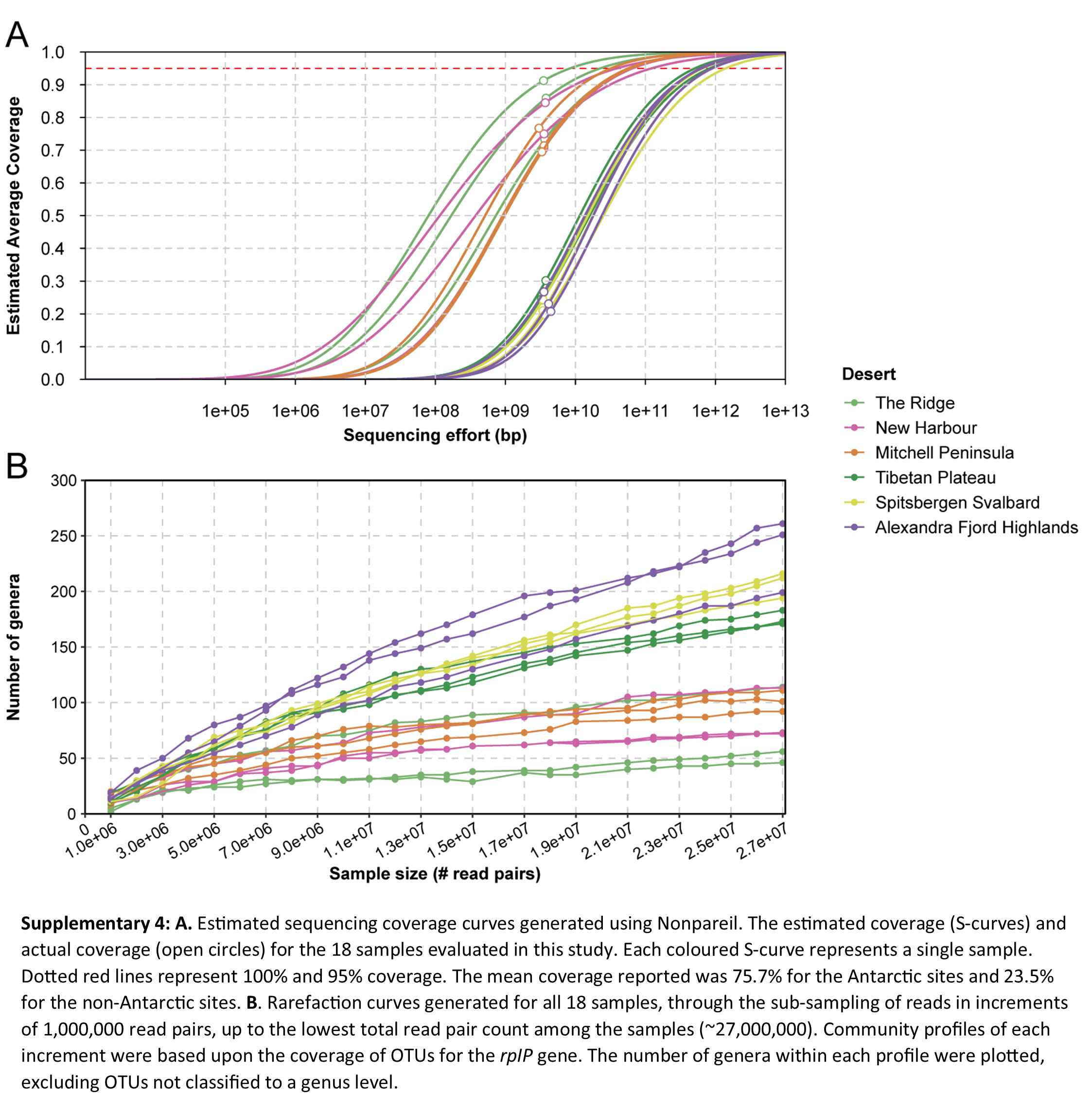

Supplement: Supplementary file 1 — Supplementary 4 [file 41396_2022_1298_MOESM1_ESM.tif]

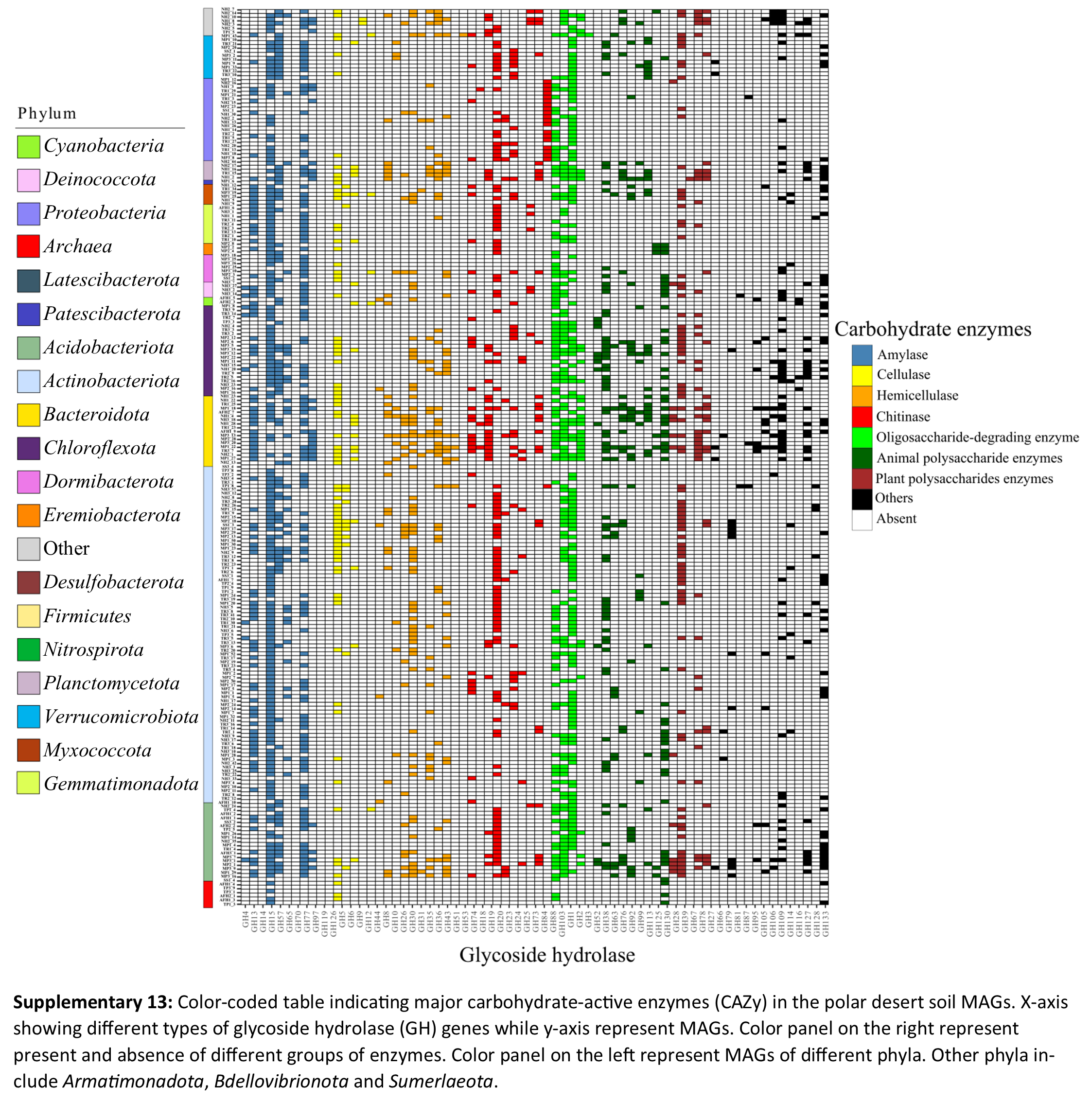

Supplement: Supplementary file 2 — Supplementary 13 [file 41396_2022_1298_MOESM2_ESM.png]

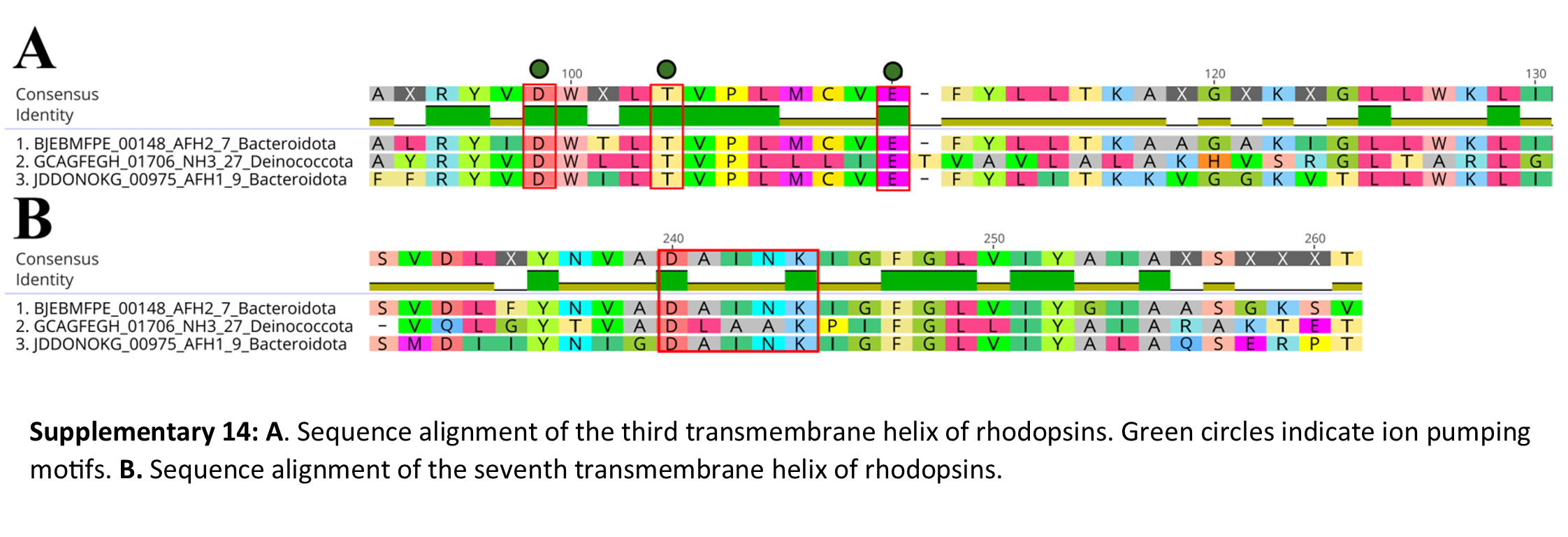

Supplement: Supplementary file 3 — Supplementary 14 [file 41396_2022_1298_MOESM3_ESM.png]

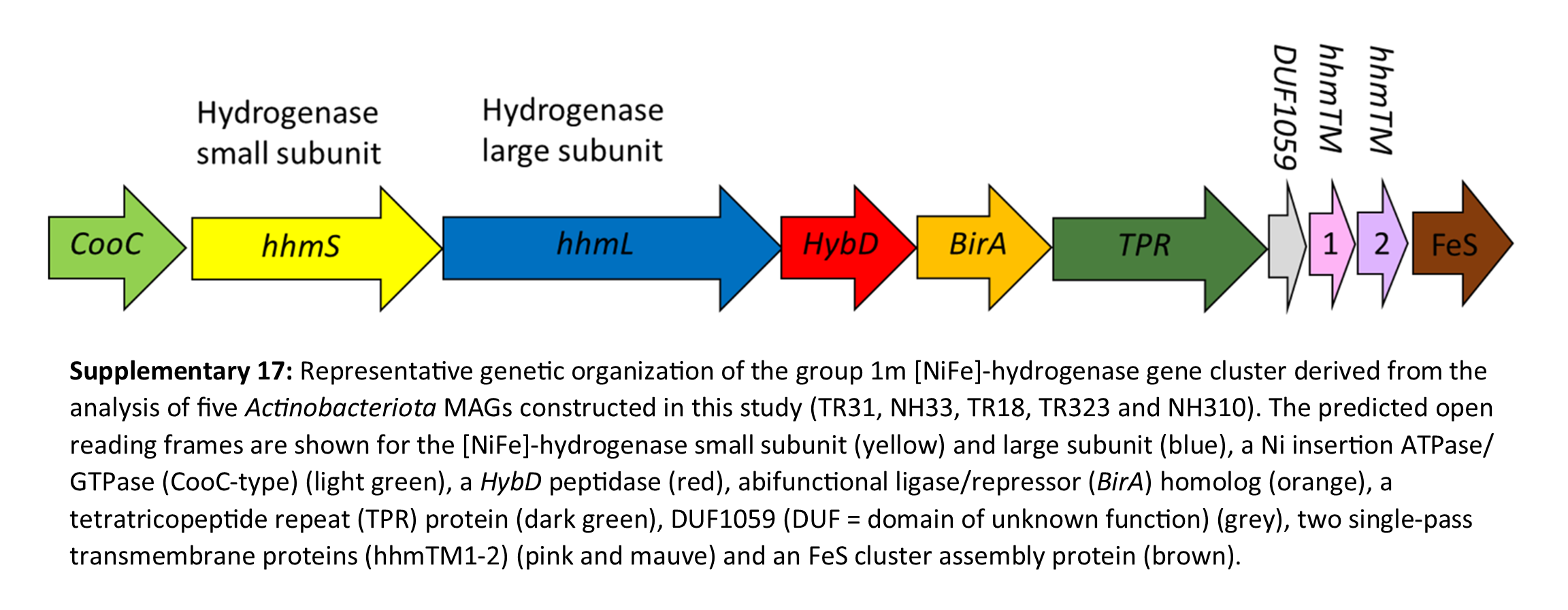

Supplement: Supplementary file 5 — Supplementary 17 [file 41396_2022_1298_MOESM5_ESM.tif]

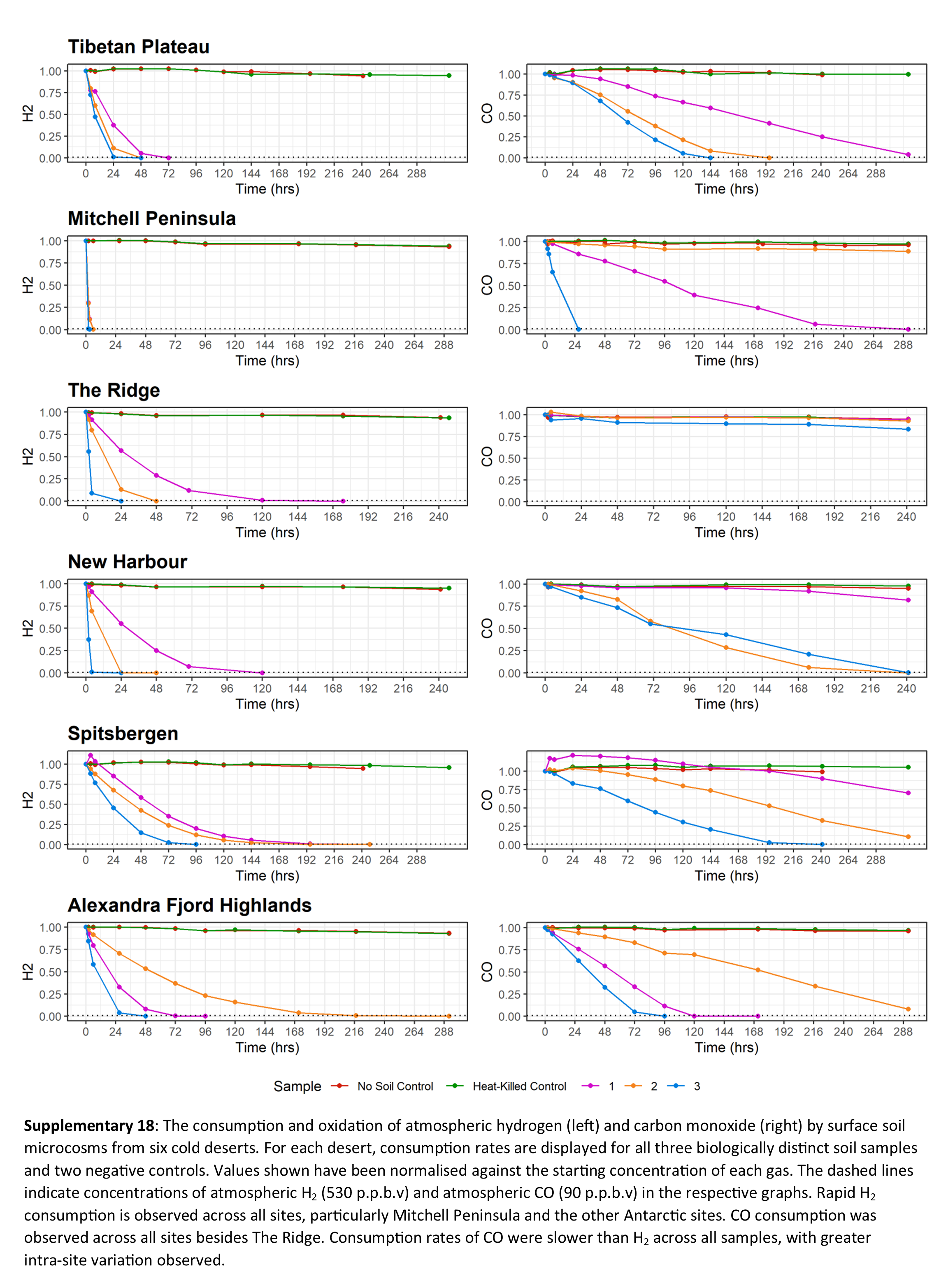

Supplement: Supplementary file 6 — Supplementary 18 [file 41396_2022_1298_MOESM6_ESM.png]
