# Supplementary material for: Atmospheric chemosynthesis is phylogenetically and geographically widespread and contributes significantly to carbon fixation throughout cold deserts
Source: ISME J. 2022 Aug 6;16(11):2547–60. doi: 10.1038/s41396-022-01298-5 (PMC9561532; doi:10.1038/s41396-022-01298-5)
Supplement: Supplementary file 4 — Supplementary 16 [file 41396_2022_1298_MOESM4_ESM.pdf]

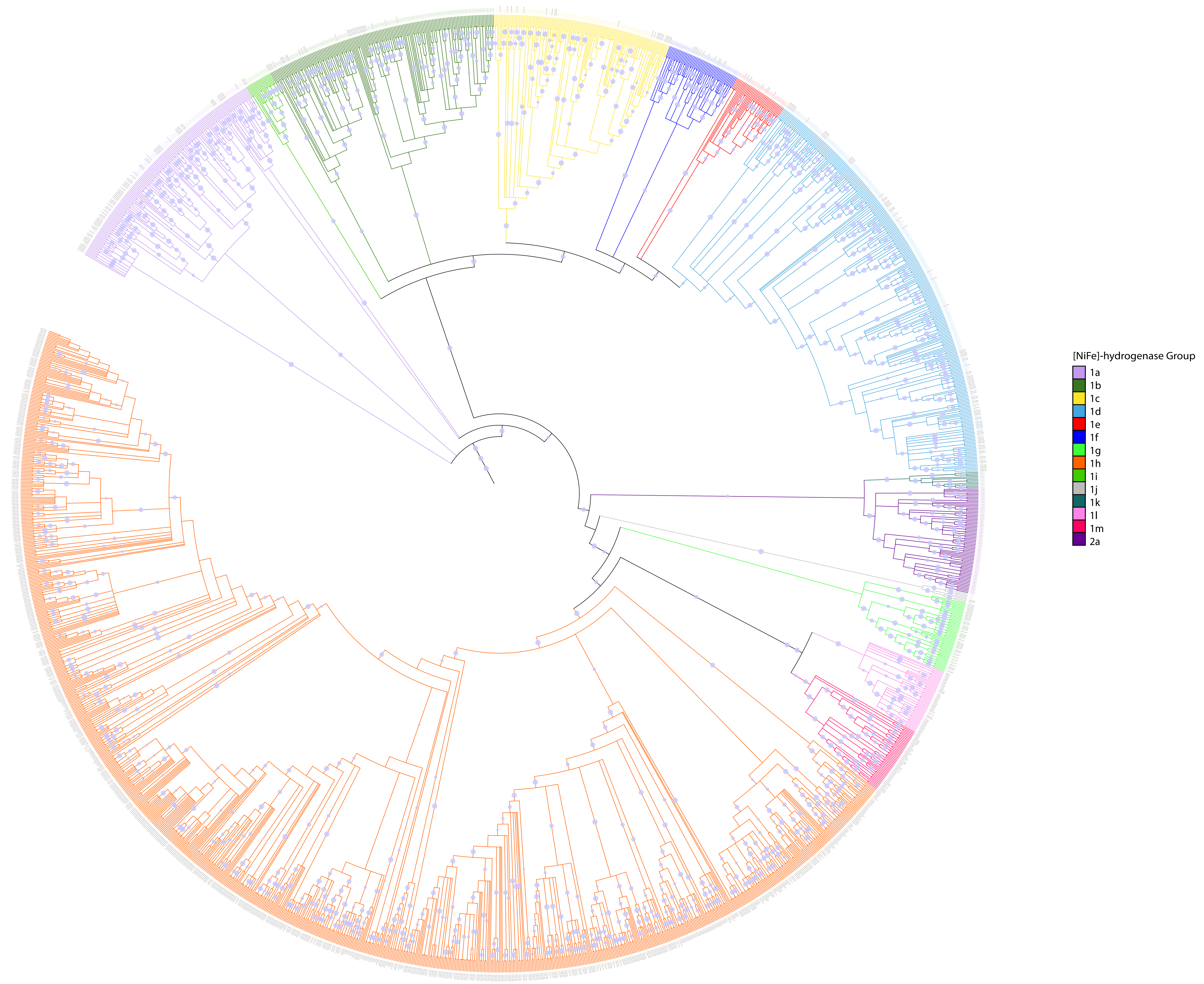

Supplementary 16: Maximum likelihood phylogenetic tree of [NiFe]-hydrogenase gene sequences obtained from our MAGS (n=304) and representative genomes from the GTDB. Branches and reference gene labels are coloured according to the group of [NiFe]-hydrogenase, including the novel group 1m [NiFe]-hydrogenase described in this paper (magenta). Bootstrap values >90% are depicted as filled circles on branches; 1000 ultrafast bootstrap iterations were applied.
