# Supplementary material for: Atmospheric chemosynthesis is phylogenetically and geographically widespread and contributes significantly to carbon fixation throughout cold deserts
Source: ISME J. 2022 Aug 6;16(11):2547–60. doi: 10.1038/s41396-022-01298-5 (PMC9561532; doi:10.1038/s41396-022-01298-5)
Supplement: Supplementary file 7 — Supplementary 19 [file 41396_2022_1298_MOESM7_ESM.pdf]

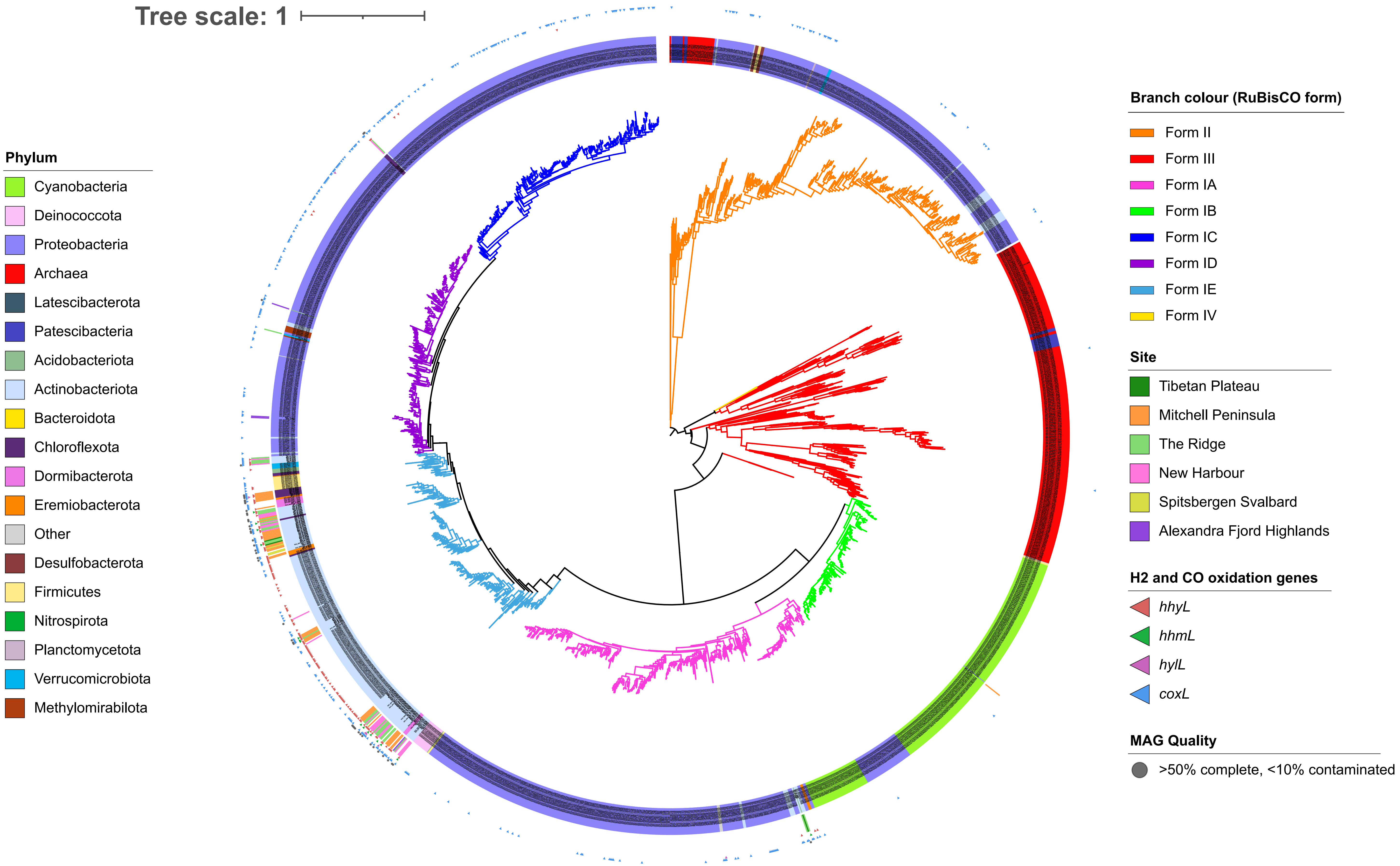

Supplementary 19: Maximum likelihood phylogenetic tree of RuBisCO gene sequences obtained from our MAGs and over 3000 published genomes. Leaves are coloured to represent phylum, while coloured branches show RuBisCO form. MAGs assembled in this study are classified according to the site where they are most abundant, and our medium to high quality MAGs are indicated by a grey outer circle. Genomes which harboured additional trace gas oxidation genes, including the high affinity groups 1h-[NiFe]-hydrogenase (*hhyL*), 1m-[NiFe]-hydrogenase (*hhmL*), 1l-[NiFe]-hydrogenase (*hylL*) and/or aerobic carbon monoxide dehydrogenase (*coxL*) with an active-site loop are indicated by outer triangles, coloured red, green, pink, and blue, respectively. Bootstrap values >90% are depicted as filled circles on branches; 1000 ultrafast bootstrap iterations were applied.
