# Supplementary material for: Atmospheric chemosynthesis is phylogenetically and geographically widespread and contributes significantly to carbon fixation throughout cold deserts
Source: ISME J. 2022 Aug 6;16(11):2547–60. doi: 10.1038/s41396-022-01298-5 (PMC9561532; doi:10.1038/s41396-022-01298-5)
Supplement: Supplementary file 8 — Supplementary 21 [file 41396_2022_1298_MOESM8_ESM.docx]

**Supplementary Methods**

**Hydrogen Oxidation Assay**

Soil samples from each region (2g) were added to separate, sterile 114 mL serum bottles and sealed with butyl rubber stoppers. Hydrogen and carbon monoxide gas were added to the otherwise ambient headspace to achieve initial concentrations of ~10 ppmv and the bottles were subsequently incubated at 10˚C. Headspaces were periodically subsampled (2 ml) using a gas-tight syringe (VICI Precision Sampling) and the partial pressure of hydrogen and carbon monoxide quantified using a pulsed discharge helium ionization detector (model TGA-6791-W-4U-2, Valco Instruments Company Inc.). As described by Islam et al (2019), this analyser quantifies hydrogen and carbon monoxide gas over six orders of magnitude. Soils from each region were tested in biological triplicate, with each Antarctic and Arctic sample selected from across the 200m sampling transect (section 2.2.). Concentrations of H_2_ and CO in each sample were regularly calibrated against ultra-pure H_2_ and CO gas standards of known concentrations, resulting in a hydrogen detection limit of 42 ppbv and a carbon monoxide detection limit of 9 ppbv. Changes in headspace hydrogen concentrations were confirmed to result from biological activity through the parallel monitoring of a heat-killed soil sample (121 °C, 15 p.s.i., 20 min) from each site and a serum bottle containing no soil. Data produced was visualised in the R v3.5.1 environment using the ggplot2 package (ver. 3.3.2) (Wickham 2016). The average rate of hydrogen oxidation was calculated for samples from each region using the first-order rate constants observed experimentally and the concentration of atmospheric hydrogen (530ppb) (Novelli et al., 1999).

**CO_2_ Fixation Assay**

Briefly, each soil sample (0.25g) was added to 4 separate sterile 4 ml glass vials and sealed with a rubber septum. Gaseous ^14^CO_2_ (1% v/v) was generated by combining 5 μl radiolabelled sodium bicarbonate solution (NaH14CO3, Perkin Elmer, 53.1 mCi nmol−1) with 75 μ l10% HCl solution, and then injected into the headspace of each vial using a gas-tight syringe to obtain a mixing ratio of 400 ppmv and otherwise ambient air. To two of the four vials, H_2_ was also added to achieve a mixing ratio of 100 ppmv. Sample groups containing ^14^CO_2_ and H_2_, and only ^14^CO_2_ were then incubated under both light and dark conditions at 10℃ for 96h. To remove any unassimilated ^14^CO_2_ prior to quantification, samples were transferred into 12 ml scintillation vials with 2 ml 10% acetic acid in ethanol and left for 12h at 30℃ to dry. Scintillation cocktail (10ml) (EcoLume) was added to each vial. A liquid scintillation spectrometer (Tri-Carb 2810 TR, Perkin Elmer) was used for radioisotope analysis, operating at 95% efficiency. Internal calibration standards were used to correct background chemiluminescence and luminescence. Negative controls were run with a heat-killed soil sample from each region. Scintillation counts from heat-killed controls were subtracted and the amount of ^14^CO_2_ fixed per sample was calculated. From each region, three separate biological samples were analysed, each in technical triplicate. Each biological sample was identical to those analysed during the hydrogen oxidation assay (section 2.10). Outliers within the technical triplicates were discarded and the average ^14^CO_2_ assimilation of each sample calculated. Data analysis and visualisation were carried out in the R environment (R Core Team, 2018) using the ggplot2 package (Wickham, 2016). For samples from each region, the variation in the levels of ^14^CO_2_ assimilated under different incubation conditions were evaluated for statistical significance using stats packages native to the R environment. Shapiro-Wilk tests were used to determine normality. Paired t-tests were applied when data distributions were normal otherwise Wilcoxon signed rank tests with Bonferroni corrections were implemented.
